# Recurrent Dermatofibrosarcoma Protuberans Over the Face: A Challenging Clinical Problem

**DOI:** 10.7759/cureus.72149

**Published:** 2024-10-22

**Authors:** Martin E Palaparthi, Sri Hari Rao Battalapalli, Munitheja Singamala, Sarmista Roy

**Affiliations:** 1 General Surgery, Sri Venkateswara Institute of Medical Sciences, Tirupati, IND; 2 General Surgery, Maulana Azad Medical College, New Delhi, IND

**Keywords:** adjuvant chemotherapy, adjuvant radiotherapy, dermatofibrosarcoma protuberans, facial tumors, forehead pedicled flap, radiotherapy, skin cancer, soft tissue tumor, wide local excision

## Abstract

Dermatofibrosarcoma protuberans (DFSP) is a rare, locally aggressive skin malignancy known for its high recurrence rate. It predominantly affects the trunk and extremities but can occasionally present on the face.

A 47-year-old man presented with a rapidly progressive, painless swelling on the left side of his face for six months. The swelling, measuring 5x4x2 cm, was well-defined, bosselated, mobile, and firm. MRI revealed a well-defined subcutaneous lesion in the left infraorbital and premaxilla regions, measuring 1.8x3.2x4 cm, with iso to hypointense signals on T1 and hyperintense on T2. Core needle biopsy confirmed DFSP. The patient underwent wide local excision with a pedicled forehead flap. Histopathology confirmed DFSP. The patient received adjuvant radiotherapy (2 Gy daily for five cycles, totaling 60 Gy) and has been disease-free for one year.

Facial DFSP is rare and presents unique diagnostic and management challenges. Early diagnosis and comprehensive surgical excision are crucial. Adjuvant therapies and advanced reconstructive techniques significantly improve outcomes. Continued research and documentation of rare DFSP cases are essential to enhance understanding and treatment strategies.

## Introduction

Dermatofibrosarcoma protuberans (DFSP) is a low- to intermediate-grade cutaneous sarcoma known for its tendency to invade surrounding tissues through local extension [1,2]. It primarily occurs on the trunk and proximal extremities, with a distribution among reported cases as follows: 42% on the trunk, 21% on the lower extremities, 21% on the upper extremities, 13% on the head and neck, and 1% in the genital area [3]. Though rare, DFSP can also arise within existing scars or tattoos [4].

DFSP is characterized by its slow-growing nature, often developing over several years [5]. This gradual growth, combined with the rarity of the condition and its diverse clinical presentations, frequently results in delayed diagnosis. Facial involvement is exceptionally uncommon and is associated with higher rates of recurrence [6]. Surgical management in these cases presents significant aesthetic challenges and requires meticulous preoperative planning. A multi-disciplinary team, including experts in surgical oncology, plastic surgery, medical oncology, radiation oncology, and anesthesia, is essential for optimal outcomes.

## Case presentation

**Figure 1:**
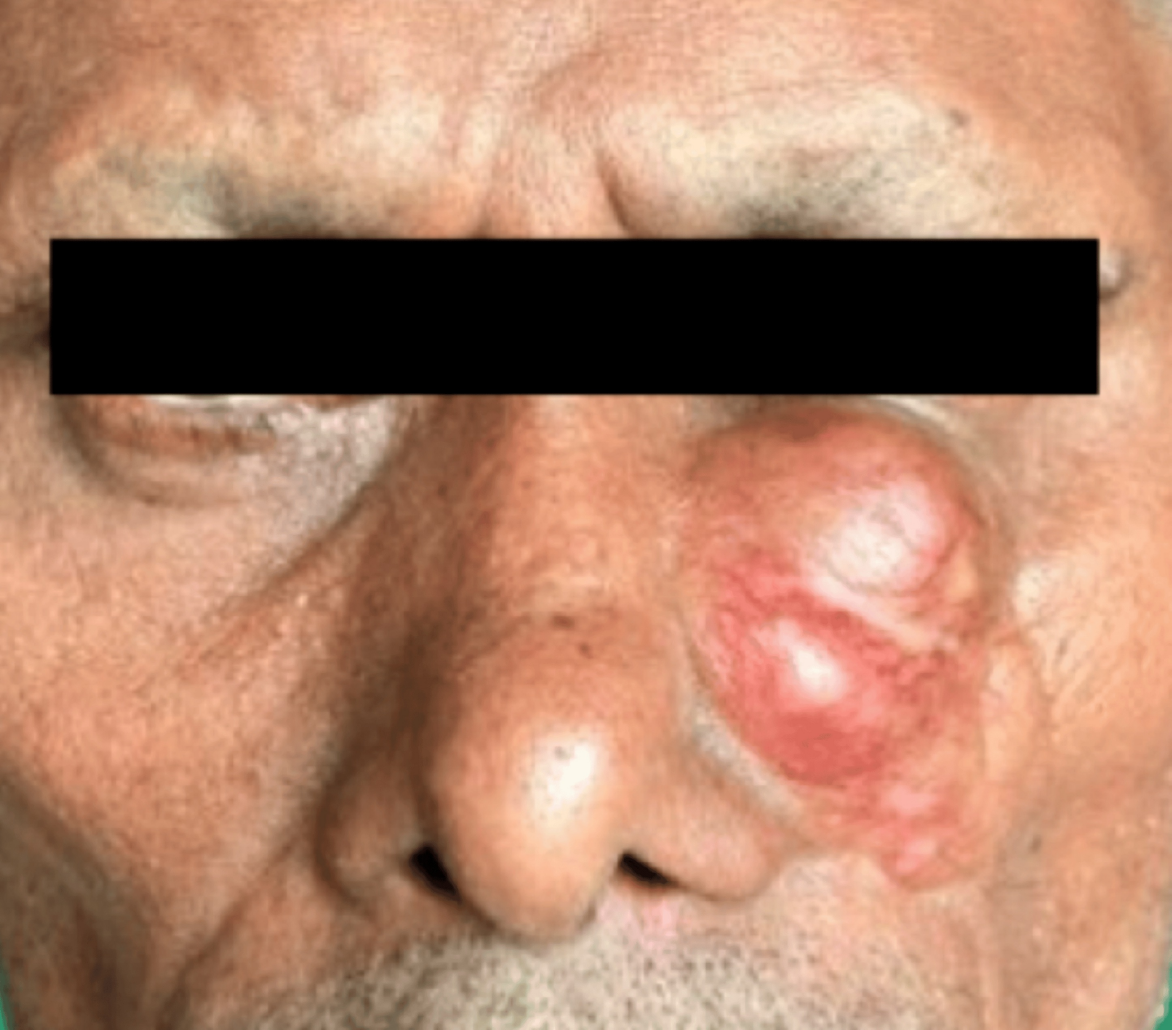
Clinical picture of the lesion

A 47-year-old man presented with a progressively enlarging, painless swelling on the left side of his face over the past six months. The swelling, which initially grew slowly, accelerated in size over the last two months. He had a history of a similar swelling at the same site, which was excised a year prior, though documentation was unavailable. Upon examination, a solitary, well-defined swelling measuring 4x5x2 cm was found in the left infraorbital region. The swelling was bosselated, mobile, and firm as shown in Figure 1. He had no signs of cervical lymphadenopathy or clinical evidence of distant metastasis.

**Figure 2:**
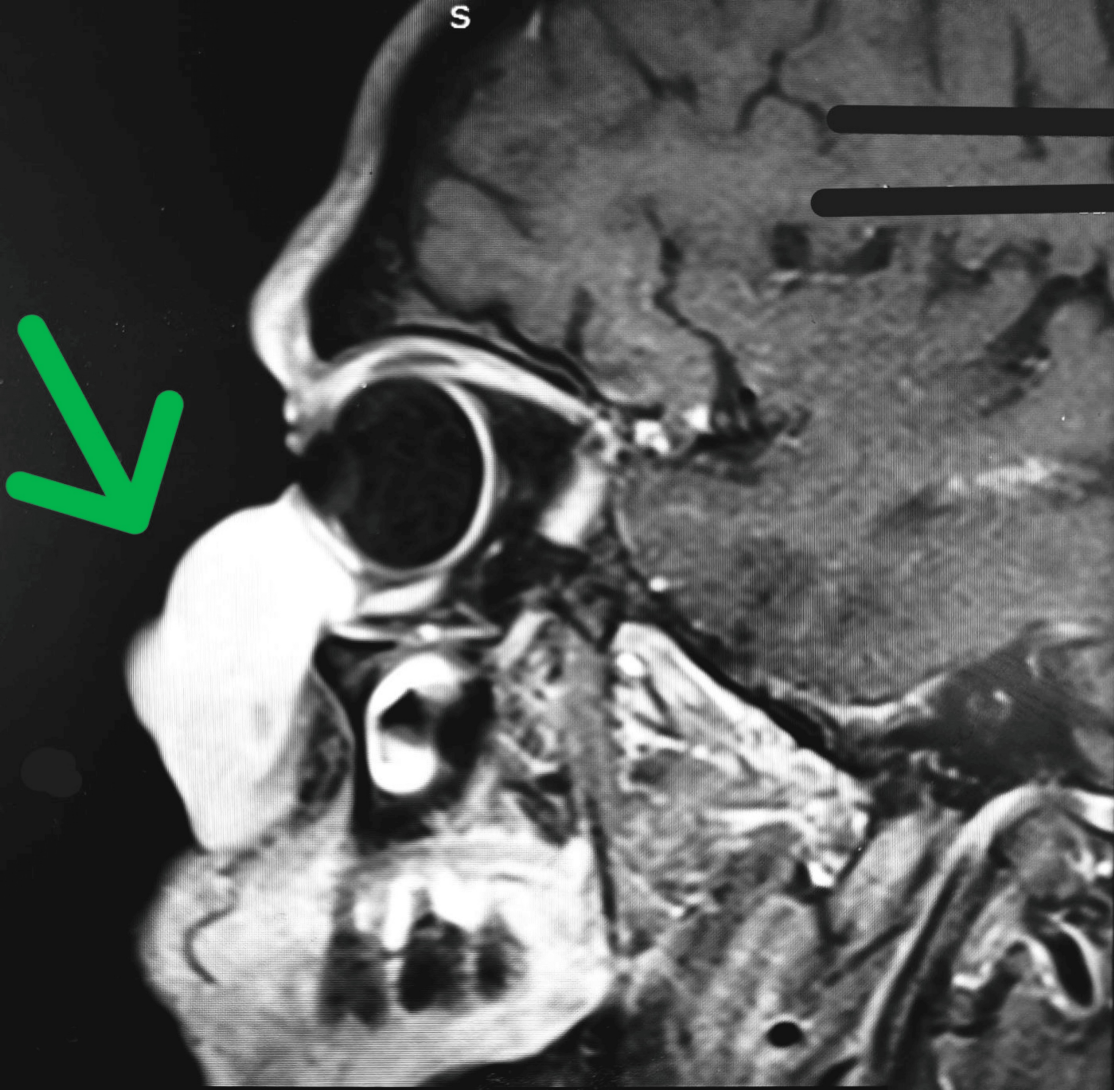
Sagittal view of MRI of the lesion indicated by the green arrow

**Figure 3:**
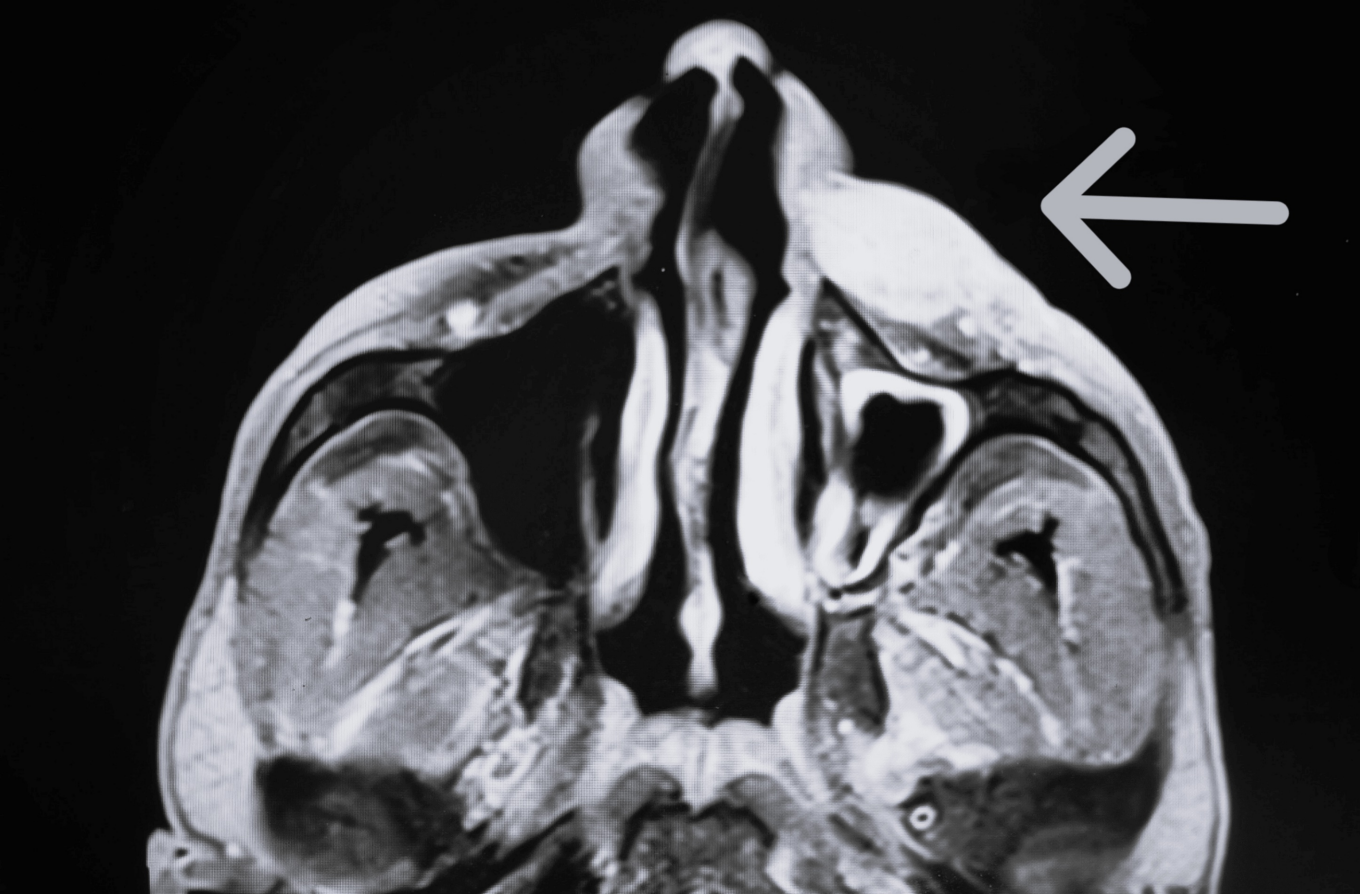
Axial view of MRI of the lesion indicated by the white arrow

MRI imaging revealed a well-defined lesion in the subcutaneous plane of the left infraorbital and premaxilla regions, measuring 1.8x3.2x4 cm as shown in Figure 2 and Figure 3. The lesion was iso to hypointense on T1-weighted images and hyperintense on T2-weighted images, with no evidence of flow voids or bony erosions.

**Figure 4:**
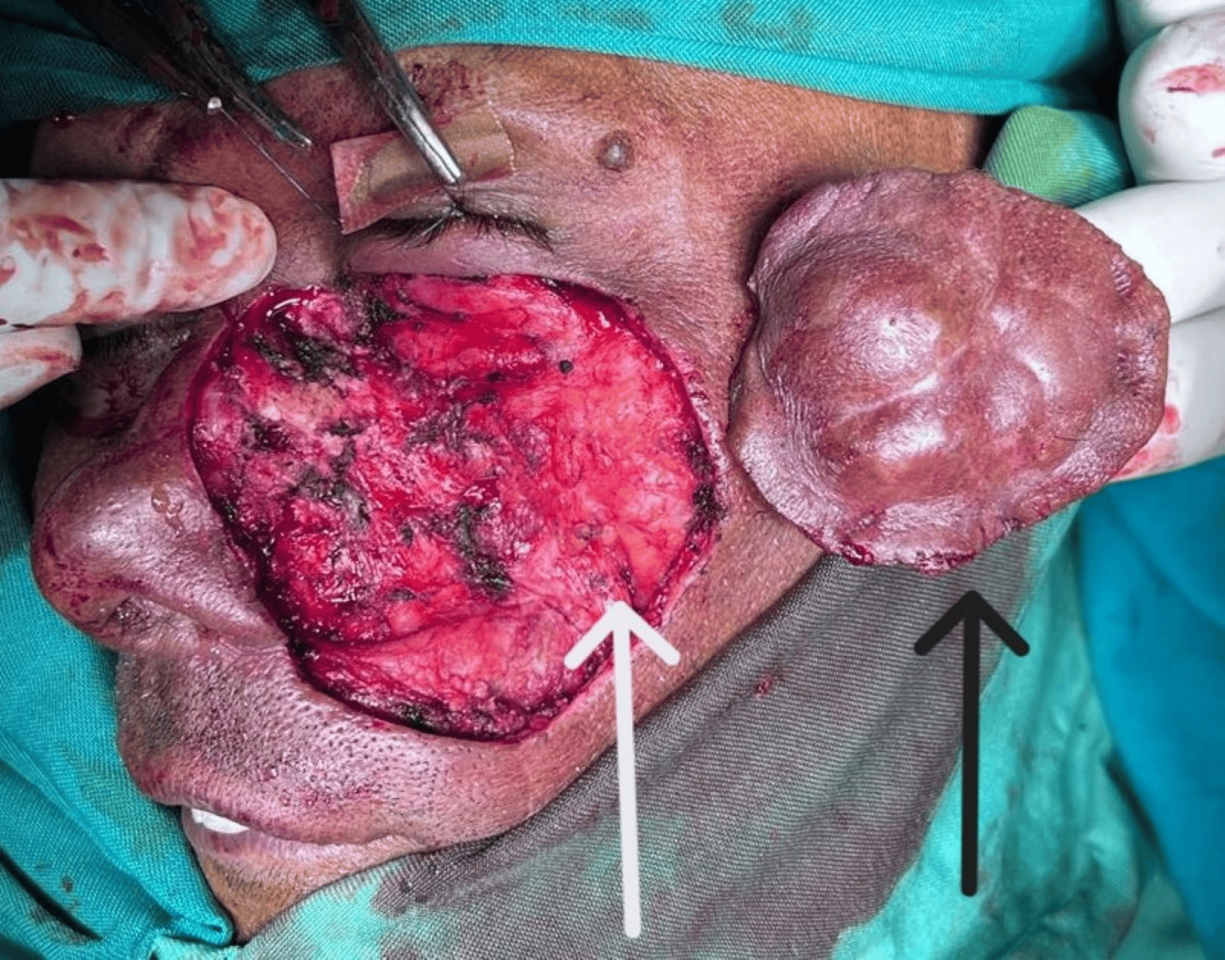
Intraoperative picture: the black arrow indicates the resected specimen and the white arrow indicates the area that needs flap coverage

**Figure 5:**
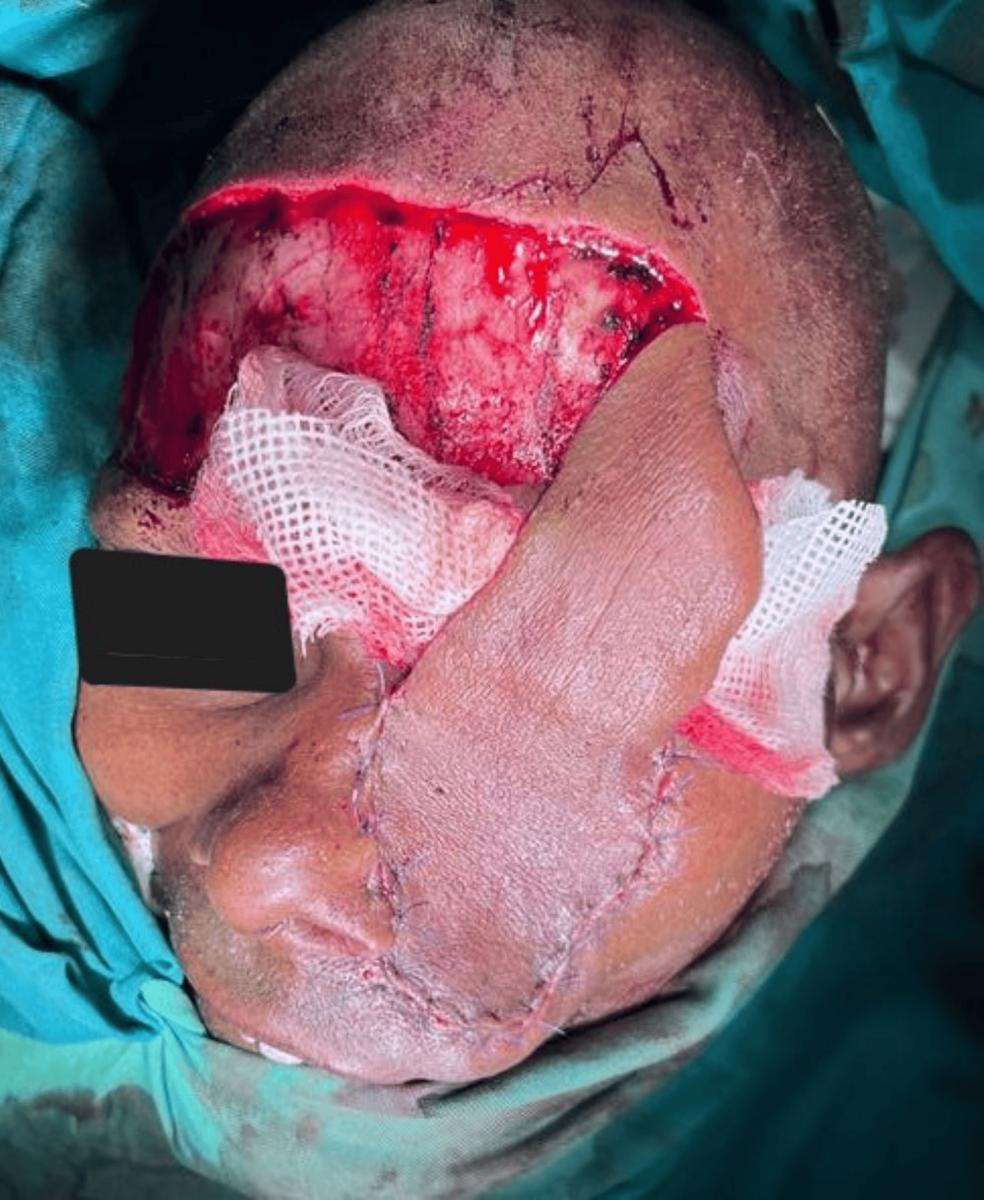
Intraoperative picture of the pedicled forehead flap

Core needle biopsy showed sheets of spindle cells with oval nuclei and scant cytoplasm intermixed with mature adipose tissue in a pale pink to mildly hemorrhagic background. The surgical procedure included a wide local excision with a 1 cm margin, as demonstrated in Figure 4. For the reconstruction, a pedicled forehead flap was utilized, as illustrated in Figure 5 and later divided. The intraoperative frozen section showed negative margins for the tumor.

**Figure 6:**
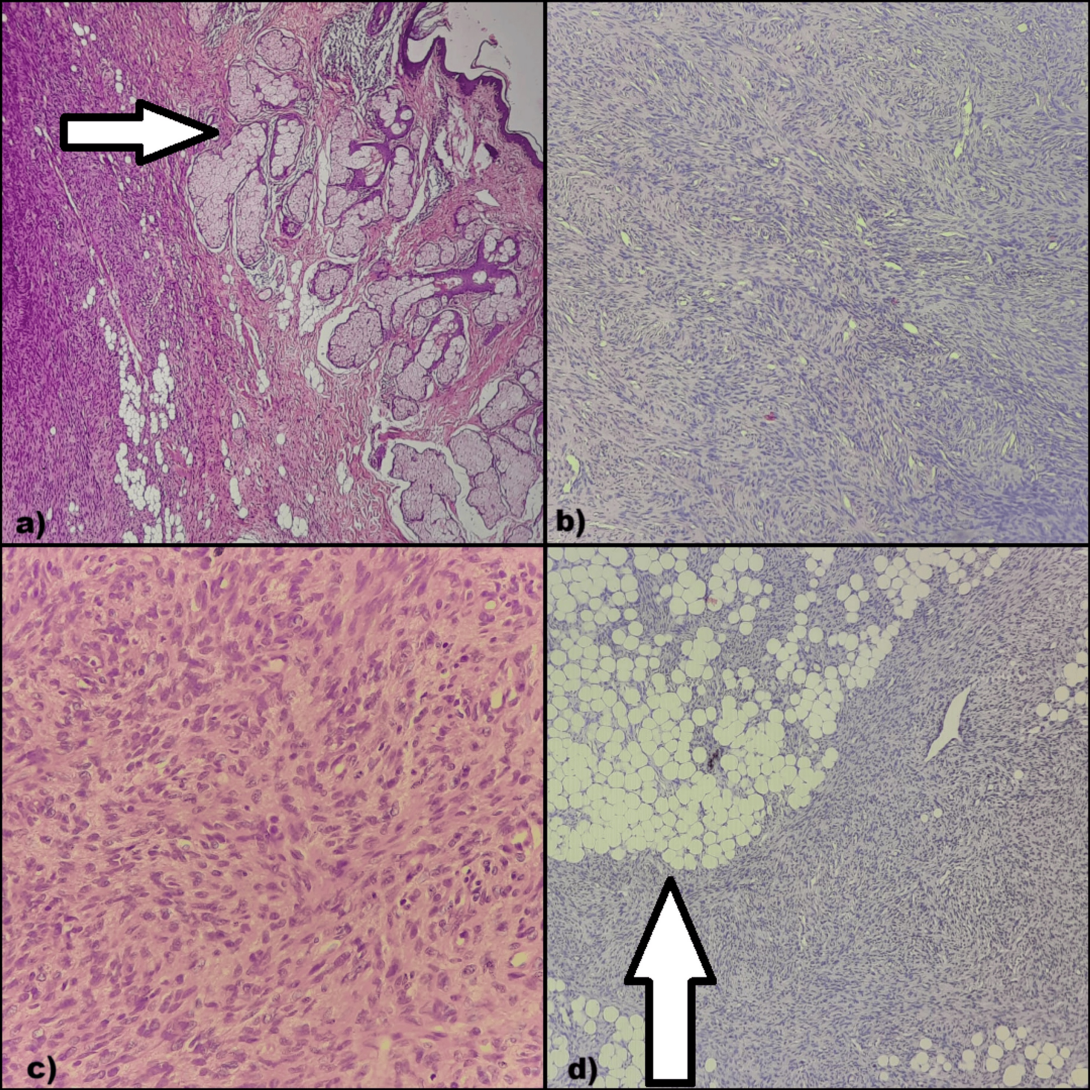
Histological findings: (a) Tumor cells seen in association with the dermis, epidermis, and skin adnexa. (b) Spindle-shaped cells arranged in a storiform pattern. (c) Spindle cells at 400x magnification. (d) Tumor infiltration into the surrounding adipose tissue.

Histopathology confirmed DFSP, characterized by spindle-shaped cells arranged in fascicles with a storiform pattern and occasional herringbone pattern as seen in Figure 6. The cells infiltrated adjacent adipose tissue and skeletal muscle.

**Figure 7:**
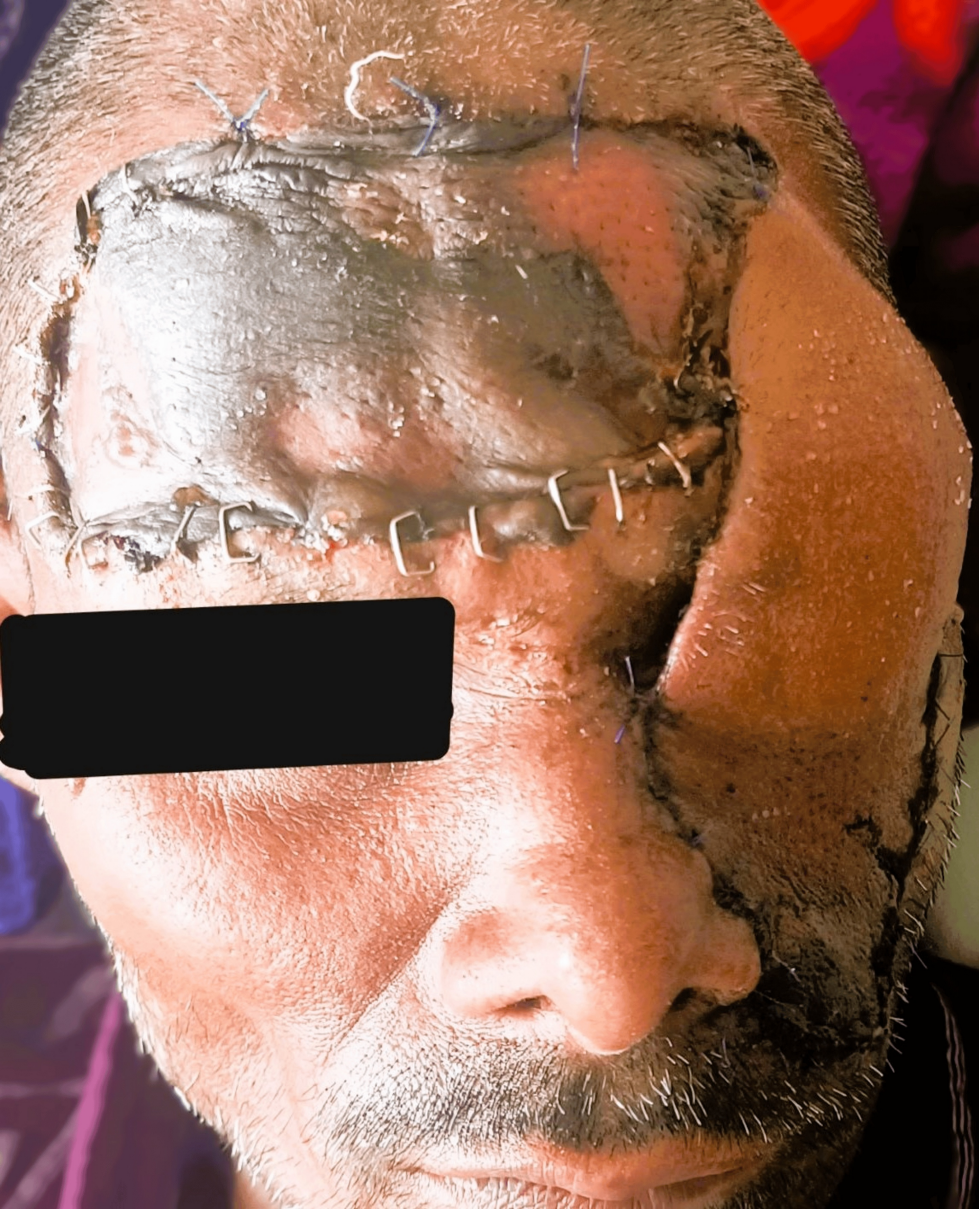
Postoperative day 6 picture showing the forehead pedicled flap and the split skin graft over the forehead

Figure 7 shows the immediate postoperative picture of the pedicled flap which was healthy and later divided.

**Figure 8:**
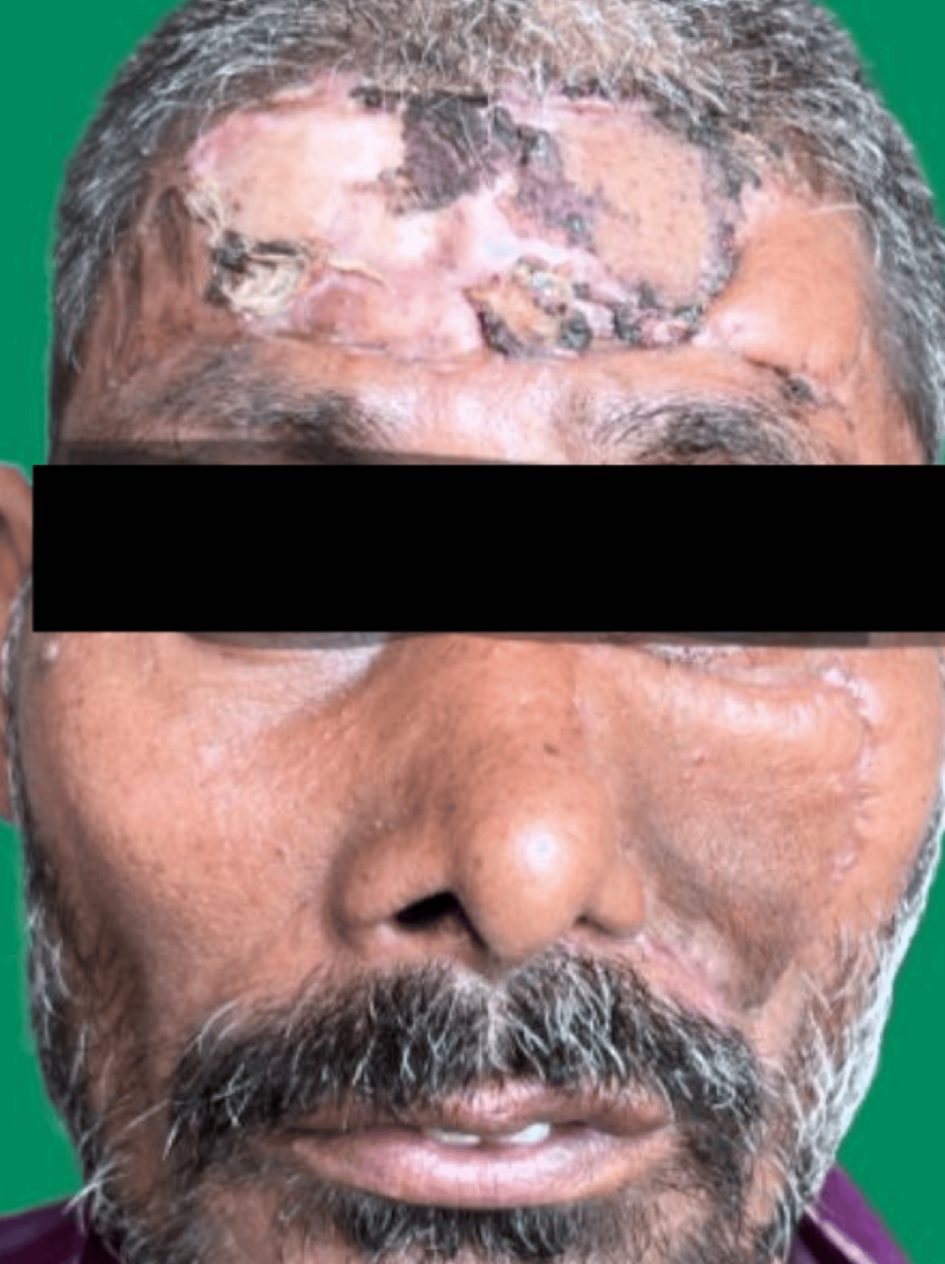
Postoperative follow-up picture

Postoperatively, the patient received adjuvant radiotherapy (2 Gy daily for five cycles, totaling 60 Gy) and had an uneventful course. At one-year follow-up, there were no signs of tumor recurrence, and the patient resumed normal activities without limitations. Figure 8 depicts the patient six weeks postoperatively, showcasing healed scars.

## Discussion

DFSP is a rare, locally aggressive cutaneous soft tissue sarcoma with a propensity for local recurrence, though metastasis is infrequent, occurring in fewer than 5% of cases. The incidence of DFSP in the United States ranges from 0.8 to 4.5 cases per million persons annually, making up 1 to 6% of all soft tissue sarcomas and 18% of cutaneous soft tissue sarcomas [7-10]. DFSP primarily affects adults in their thirties but can occur in any age group, including children and older adults, with a slight male predominance [11,12].

DFSP typically presents as an indurated, asymptomatic plaque that slowly enlarges over months to years. The tumors often have a skin color varying from brown-yellow to red-tinged, with early lesions possibly showing violet-red or blue margins. As the tumor grows, it becomes firm and nodular, sometimes with telangiectasia [5]. In advanced stages, the tumor can ulcerate, bleed, or become painful, and may reach large sizes if untreated. At diagnosis, most DFSP tumors are superficial and less than 5 cm in diameter [13].

DFSP should be considered in patients with a history of a firm, slow-growing cutaneous nodule. Dermoscopy may suggest DFSP, but a core needle or incisional biopsy is essential for a definitive diagnosis. Grossly, DFSP appears as a white to yellow, poorly circumscribed mass with a fish flesh-like texture. Histologically, DFSP originates from dermal fibroblasts, showing spindle cells with marked cellular and nuclear pleomorphism. Immunohistochemically, DFSP spindle cells express CD34 strongly and diffusely, while other markers like alpha-smooth muscle actin and S-100 are negative [14].

Magnetic resonance imaging (MRI) shows DFSP as a well-defined, hypointense mass on T1-weighted images and heterogeneous hyperintensity on T2-weighted images. Gadolinium enhancement is moderate to intense, reflecting the tumor’s vascularity. Computed tomography (CT) shows variable attenuation depending on the tumor’s composition and is useful for assessing bone involvement. PET-CT aids in staging, monitoring treatment response, and detecting recurrence [15]. Molecular diagnostics can detect the COL1A1-PDGFB gene fusion by FISH or RT-PCR, which is crucial for diagnosis and treatment guidance, though it may be absent in about 8% of cases [16].

Surgical excision is the standard treatment for DFSP, with options including wide local excision (WLE) and Mohs micrographic surgery (MMS). MMS is often preferred due to its lower recurrence rate compared to WLE [17]. Adjuvant radiation therapy is effective for controlling growth and preventing recurrence in cases of incomplete excision or inoperable tumors. The typical radiation dose is 60 Gy for microscopic tumors and 70 Gy for macroscopic tumors [18].

Imatinib mesylate (IM) targets the PDGFR pathway and is effective for inoperable, locally recurrent, or metastatic DFSP. It induces tumor shrinkage but is effective in only about 90% of cases. Neoadjuvant IM can make tumors operable, and surgical resection is recommended after IM treatment to confirm response and prevent resistance. Sunitinib is an alternative for IM-resistant cases [19].

Postsurgical resection with negative margins generally leads to favorable outcomes, with five-year disease-free survival rates of around 86%. Poor prognostic factors include older age, high mitotic index, positive margins, and increased cellularity. Local recurrence occurs in 20% to 50% of cases, especially within the first three years post-treatment, necessitating close follow-up [20]. Regular evaluations should include thorough history, clinical examination, and imaging as needed.

## Conclusions

DFSP is a rare, locally aggressive soft tissue sarcoma that requires prompt recognition and management due to its potential for recurrence. Histopathological examination with immunohistochemical staining is vital for accurate diagnosis, and Mohs micrographic surgery remains the gold standard for minimizing recurrence risk through precise margin control. In cases where surgical margins are insufficient or the tumor is inoperable, adjuvant therapies such as radiotherapy and targeted treatments like imatinib play a crucial role. Early detection and a multidisciplinary approach are essential for optimal patient outcomes and improved quality of life. Long-term active surveillance is critical, given the potential for local recurrence, particularly in challenging anatomical regions like the face.
